# Do Cash Transfer Programmes Affect Child Anaemia? Results From a Meta‐Analysis

**DOI:** 10.1111/mcn.70026

**Published:** 2025-03-28

**Authors:** James Manley, Harold Alderman

**Affiliations:** ^1^ Economics Department Towson University College of Business & Economics Towson Maryland USA; ^2^ International Food Policy Research Institute Washington, DC USA

**Keywords:** anaemia, cash transfers, haemoglobin, social protection

## Abstract

Childhood anaemia is common and debilitating. Nutrition‐specific policies are effective for addressing anaemia in many contexts but less is known about nutrition‐sensitive policies such as cash transfers. We reviewed over 4000 studies and gathered 26 estimates of the effect of cash transfer programmes on childhood haemoglobin and anaemia. Overall, neither the impact of cash on haemoglobin (0.065 d/L, CI [−0.054, 0.184]) nor on anaemia prevalence (−0.092, CI [−1.227, 1.042]) were significant. While cash on its own had basically a null effect, programmes that provided cash in combination with other interventions such as behaviour change communication or nutritional supplements were more successful. The impact of social protection on haemoglobin and anaemia is surprisingly understudied compared to height, on which a previous study found well over 100 impacts of cash transfer programmes. Overall impacts of cash transfer programmes on haemoglobin and anaemia are weak: evidence is inconclusive at best. Cash transfer programmes are more successful in combination with other programmes providing education and/or nutritional supplements.

## Summary

- We conducted a broad search of the literature on cash transfers and child health, locating 26 estimates of programme impacts on haemoglobin and/or anaemia.
- Meta‐analysis found that estimated programme impacts were not statistically different from zero, but meta‐regression analysis showed that programme characteristics such as nutritional supplements and access to health care predicted improvements in Hb and anaemia.
- Surprisingly few studies include anaemia or haemoglobin as outcomes of interest. More research could help improve programme effectiveness.

## Graphical abstract

We gathered 26 estimates from 4000 studies of cash transfer programmes' impacts on childhood haemoglobin and anaemia prevalence. Effects of cash alone on either outcome were insignificant, but programmes that provided cash in combination with other interventions such as health care, behaviour change communication, or nutritional supplements were more successful.

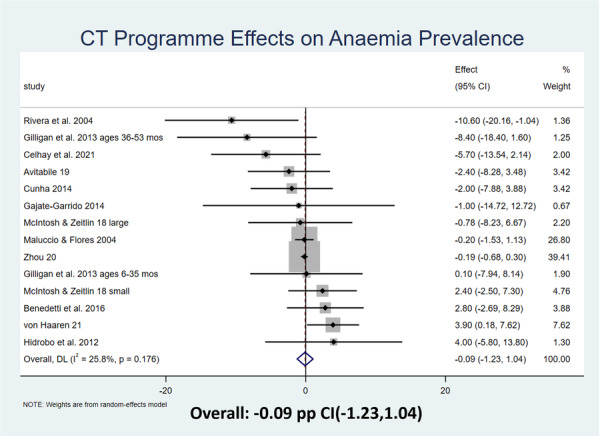

## 1 Introduction

Sustainable Development Goal 2.2 calls for ending all forms of malnutrition. To achieve this the world will have to address anaemia, which is the most prevalent nutritional problem globally (Balarajan et al. 2011). Progress on reducing anaemia will also contribute to improved cognitive, motor, and socioemotional development, as well as work capacity all of which are associated with anaemia or low levels of haemoglobin (Hb). While a fair share of this global burden is manifested in risks during pregnancy as well as in worker productivity, anaemia is also a concern for children aged 6–59 months. As of 2019, the prevalence of anaemia in children was about 40% (WHO 2023). Jain et al. (2024) estimate an economic burden for children's anaemia of about US$161 billion per year worldwide. In India it is the single most important nutritional risk factor, with iron deficiency anaemia among children leading to economic losses calculated at 1.3% of gross domestic product (Plessow et al. 2015).

A 2020 meta‐review of 118 systematic reviews of both nutrition‐specific and nutrition‐sensitive interventions (Moorthy et al.) found that the former are more effective against anaemia. In addition to malaria treatments and delayed cord clamping, the most effective interventions include supplementation with iron and micronutrient powders. They found that micronutrient powders were associated with increases in Hb and decreases in anaemia risk, the same conclusion drawn by Andrew et al. (2016). Moorthy et al. also found iron supplementation to be associated with a reduction in anaemia, a conclusion echoed in Gutema et al. (2023). Similarly, Keats et al. (2021) classify supplementation among the nutrition‐specific interventions with a strong evidence base on effectiveness in addressing maternal nutrition. They also find a growing evidence base for malarial prevention contributing to improved nutrition. As studies show correlations between child anaemia with maternal anaemia (Heesemann et al. 2021; Ntenda et al. 2018; Shukla et al. 2019) these programmes that are efficacious for maternal nutrition may have the potential to also influence child anaemia.

However, the review by Moorthy et al. found that there is much less evidence on the impact on Hb or anaemia from interventions that are nutrition‐sensitive such as transfer programmes as well as the promotion of agriculture and food security. One common nutrition‐sensitive intervention, cash transfers, might lead to increased quantities of foods purchased as well as improved diet quality and, thus, potentially to improved iron status. Moorthy et al. (2020) were able to review only one paper on cash transfers, Segura‐Pérez et al. (2016), which found that of the three country‐wide cash transfer programmes in Latin America that influenced child health only one, covering a programme in Mexico, found links to increased haemoglobin. In contrast, a Brazilian study in their review found a negative correlation between haemoglobin levels and participation in a cash transfer programme (Silva et al. 2021). In a systematic review of the effects of cash transfers in 129 studies, Manley et al. (2022) found an increase in animal‐source foods consumed of 6.7 percentage points and an increase in dietary diversity of about 0.6 food groups. These improvements translated to a small drop in stunting (and an increase in height for age *z*‐scores) but no increase in weight for age or wasting. This analysis, however, did not review the impacts of the improved diet on anaemia.

Improvements in diet are worth noting, but it is unclear whether an improved diet alone translates to changes in anaemia status. First, although iron deficiency is said to account for between 25% and 42% of all anaemia cases, disease is also a common contributing factor (Victora et al. 2021). Disease and poor diets can both limit haemoglobin levels, and the two pathways can also interact. As to the latter, iron intake is not directly tied to iron deficiency anaemia: the role of diet quality is complex since haemoglobin absorption depends, in part, on the composition of the diet, not only whether it includes animal‐source foods and vitamin C, but also whether absorption is inhibited by tannins in the diet as well as calcium and zinc supplements. In a summary of the role of diet in anaemia, Loechl et al. (2023) summarize, ‘Although beneficial for meeting requirements for multiple essential micro‐ and macronutrients, diet quality appears to have mixed results on iron status and bioavailability, potentially because of implementation of these interventions at the household and population level’ (p. S47). Dietrich and Schmerzeck (2019) find that cash transfers do not have much effect on nutrient availability except in some drought‐affected households in less isolated areas. In a cross‐country study, Alderman and Linnemayr (2009) found that increased income has a positive but modest impact on anaemia.

Manley et al. (2020, 2022) investigated the link between cash and diet, but they did not directly address anaemia. We start by reviewing the same literature for estimates of anaemia reduction: building on Manley et al. (2020, 2022) this review investigates the degree to which cash transfers improve haemoglobin concentration and anaemia among children under age 4. Reviewing 26 estimates from 19 studies which reported relevant outcomes we find that cash transfer programmes on average have only small, insignificant effects on haemoglobin and anaemia.

## 2 Methods

### 2.1 Data Collection

We started with data collected for Manley et al. (2020) and (2022), gathered by a review of over 4000 citations on the relationship between cash transfers and child health. The authors of Manley et al. (2022) searched using two terms combined with Boolean operators: ‘cash transfer’ and (‘child health’ or ‘child nutrition’). From the 4000+ results, abstracts were first screened, followed by full text for the outcomes, HAZ, stunting, WAZ, WHZ, wasting, animal‐source foods, dietary diversity, and diarrhoea incidence. Articles reporting impacts of cash transfer programmes on haemoglobin, iron, or anaemia among children, adolescents, or mothers were flagged during that review, and later we separated out impacts by the outcome analysed and the ages of the sampled group. In keeping with the stated objectives and targeting of many transfer programmes the vast majority of the estimates were for children under 5 years of age, so our analysis is focused there. We also were unable to gather data sufficient to warrant analysis on iron status as an outcome. Specifically, there were very few papers on iron levels (Dietrich and Schmerzeck 2019; Harris‐Fry et al. 2018; Ramírez‐Silva et al. 2013 among them), and six studies that tracked impacts on adults and older children.^1^

The inclusion/exclusion criteria used were as follows. All studies need (1) clear counterfactuals, including randomized control trials, regression discontinuity, or propensity score matching; (2) estimates of impact on targeted outcomes with information on variability of estimates sufficient to calculate confidence intervals (CIs); (3) 300 or more observations on at least one outcome; in (4) countries with per capita gross domestic product of under US$10,000 and (5) substantial literature not covered in existing meta‐analyses (Manley et al. 2022). (The last criterion refers to the decision not to gather data on outcomes such as low birthweight, which were dealt with quite completely in other studies.).

After working through the data gathered during the 2021 search, we looked for updated results in systematic reviews, including Aurino and Giunti (2022), Awojobi (2021), Innocenti UNICEF Office of Research (2022), Jeong and Trako (2022), McWay et al. (2022), Mishra and Battistin (2017), Pega et al. (2015, 2022), Tappis and Doocy (2018), Tirivayi et al. (2020) and van Daalen et al. (2022). No new papers were identified.

We also updated the search by searching ‘cash transfers’ and (haemoglobin or anemi*) in the following databases (replacing anemi* with anaemi* where appropriate). That yielded the following number of results in a search in July 2024: Agris (0), Econlit (7), IDEAS (17), IFPRI (2), PubMed (26), Scopus (64), and the World Bank's ‘open knowledge’ database (35). We found no new citations in these 151 search results. Next, we tried three searches in Google Scholar, looking in each case at the first 100 search results. We tried the above search without limiting the years of publication, with limiting the years of publication to after 2018, and separately removing the word ‘iron’. In each case we identified informative works that contributed to the literature review but no new trial results.

### 2.2 Data Analysis

Our first step is to combine the estimates meta‐analytically to get an overall average estimate of programme effectiveness. Next, we investigate other programme characteristics potentially associated with improved health. Here we follow Manley et al. (2022), employing meta‐regression analysis on several explanatory variables, one at a time. We consider transfer amounts, programme characteristics including the inclusion of behaviour change communication, access to supplements, and health‐based behaviour change communication, as well as rates for child and maternal anaemia by country and year from the World Development Indicators.

Finally, we investigated whether any study unduly influences our results. We repeat both meta‐analyses dropping one observation at a time. We also assess whether the sampled studies are biased by selective publication.

### 2.3 Data

We found enough information (i.e. point estimates and standard errors) to permit meta‐analysis on two outcomes: haemoglobin concentrations in grams per decilitre, which were reported in 12 studies, and anaemia prevalence in percentage points, generally defined as an altitude adjusted level of 11 g/dL, which shows up in 14 studies. In total we have 26 observations from 19 articles, as three articles contributed two estimates each tracking the effects of different amounts of cash or effects on children of different age groups.

Table 1 shows the results from the 14 studies that report programme impacts on anaemia (some with multiple measurements from different samples) and the 12 studies that report Hb. (See Appendix Tables A1 and A2 for more information about each of the programmes listed in Table 1.) Of the studies that look at anaemia, nine specify that they are using a cutoff of 11 g/dL to identify anaemia. Two more specify that they are following the Demographic and Health Surveys protocol, which uses the same cutoff. Just three papers: Avitabile et al. (2019), Celhay et al. (2016) and Gilligan et al. (2013) do not specify the cutoff used. Six papers specify that Hb measurements are adjusted for altitude; for the others this detail is not mentioned.

**Table 1:** List of studies.

Study	Programme	Country	Mean age of sample (years)	Haemoglobin impacts (g/dL)	Anaemia prevalence	Supplements?	Healthcare?
Avitabile et al. (2019)	PAL	Mexico	3	—	−2.4	n	n
Benedetti et al. (2016)	Bono 10,000	Honduras	1.5	—	2.8	n	y
Celhay et al. (2016)	Bono Juana Azurduy	Bolivia	< 5	—	−5.7	y	y
Cunha (2014)	PAL	Mexico	4	—	−2	n	n
Fenn et al. (2017), single cash	Action against hunger	Pakistan	2	−0.12	—	n	n
Fenn et al. (2017), double cash	Action against hunger	Pakistan	2	0.07	—	n	n
Fernald and Hidrobo (2011)	BDH	Ecuador	2	0.04	—	n	n
Fernald et al. (2008)	PROGRESA	Mexico	3.5	0.9	—	y	y
Gajate‐Garrido (2014)	Juntos	Peru	3	0.41	−1	y	y
Gilligan et al. (2013): 6–35 months	Karamoja cash transfer	Uganda	2	—	0.1	n	n
Gilligan et al. (2013): 36–53 months	Karamoja cash transfer	Uganda	3.8	—	−8.4	n	n
Hidrobo et al. (2012)	WFP Ecuador	Ecuador	2.5	−0.13	4	n	n
IFPRI International Food Policy Research Institute (2003)	PRAF	Honduras	1.5	0.1	—	nc	y
Maluccio and Flores (2004)	Red Protección Social	Nicaragua	2.5	−0.1	−0.2	nc	y
McIntosh and Zeitlin (2018), small	Based on Gikuriro	Rwanda	< 5	—	2.4	n	n
McIntosh and Zeitlin (2018), large	Based on Gikuriro	Rwanda	< 5	—	−0.78	n	n
Paxson and Schady (2010)	BDH	Ecuador	3	0.094	—	n	y
Perova and Vakis (2009)	Juntos	Peru	< 5	−0.45	—	y	y
Rivera et al. (2004)	PROGRESA	Mexico	0–1	0.37	−10.6	y	y
von Haaren and Klonner (2020)	IGMSY	India	4	—	3.9	n	y
Younger et al. (2009)	BDH	Ecuador	2	−0.13	—	n	n
Zhou et al. (2020)	MCH CCT	China	1	—	−0.19	n	y

Five programmes consistently provided supplements. The two papers (Fernald et al. 2008; Rivera et al. 2004) reviewing results of the Mexican programme, PROGRESA, that provided Nutrisano, a powdered micronutrient supplement that was added to water and given as a drink. The two papers (Gajate‐Garrido 2014; Perova and Vakis 2009) reviewing the Peruvian programme Juntos mention that vitamin A supplements were part of the original package; Gajate‐Garrido mentions that iron supplements were also provided if indicated. The supplement provided in the Bolivian programme is not mentioned in Celhay et al. (2016) but a reference is made on a website to a supplement ‘to help prevent infant anaemia’ (AIN Andean Information Network 2011).

For additional information on the studies, see Appendix Table A1, including impact estimates with confidence intervals, and Appendix Table A2, with more details about the programmes.

## 3 Results

**Figure 1:**
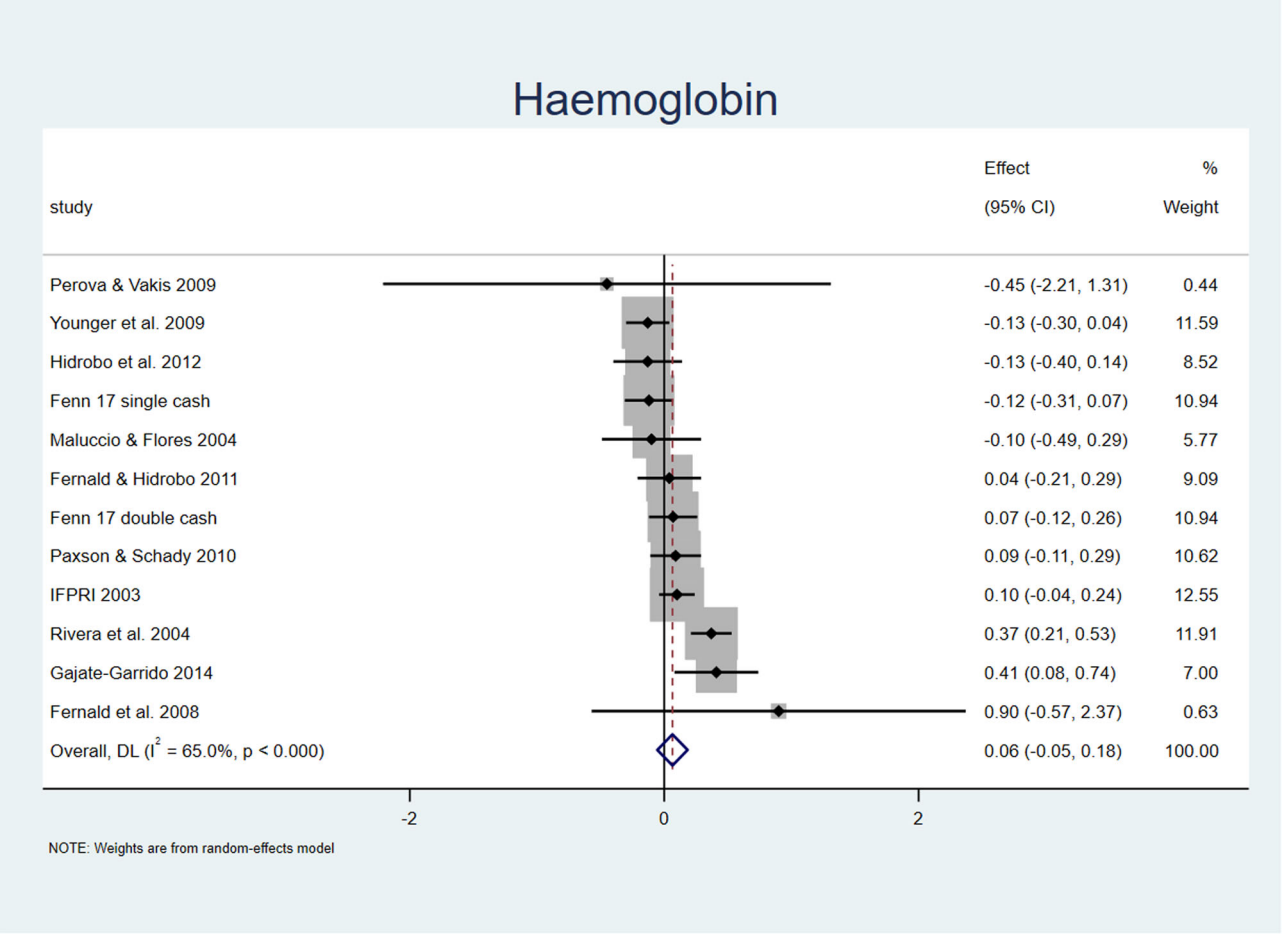
Forest plot showing programme impacts on haemoglobin, g/dL.

Figure 1 shows the average combined impact of our 12 estimated impacts of cash transfers on haemoglobin, with cash transfer programmes failing to demonstrate a statistically significant impact. The pooled impact on haemoglobin is positive but with a point estimate of only 0.065 g/dL (CI[−0.054, 0.184]), an effect statistically insignificant with a *p* value of 0.29.

**Figure 2:**
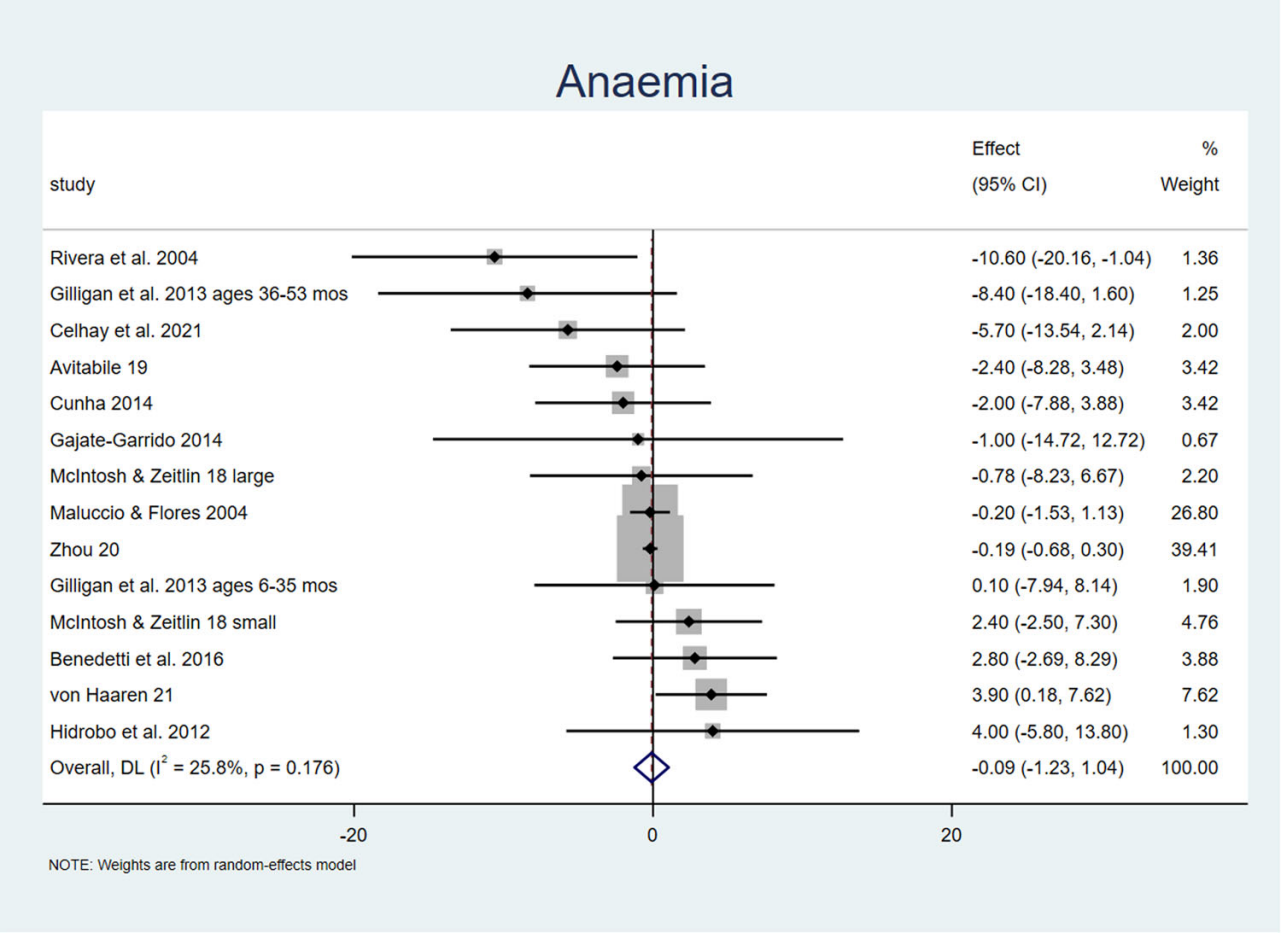
Forest plot showing programme impacts on anaemia prevalence. *Note:* Weights are from random‐effects model.

Figure 2 shows that 14 estimates on average find a small decrease in anaemia prevalence. The pooled effect is a decrease of 0.09 percentage points (CI [−1.227, 1.042]), insignificant with *p* > 0.87.

Next, we tried a robustness check, repeating both meta‐analyses but dropping one observation at a time. When we do so for haemoglobin, we find that average programme effects range from 0.019 to 0.088, with the lowest p‐value being for the highest estimate (0.088) at *p* = 0.167. When we do so for anaemia, average programme effects range from −0.285 to 0.009, with the lowest *p*‐value being for the lowest estimate (−0.285) at 0.459.

The next step is to investigate other programme characteristics potentially associated with improved health. Below are the results of our meta‐regressions, which involved each dependent variable (i.e. haemoglobin and anaemia status) regressed one at a time on a series of programme and sample characteristics. Explanatory variables included transfers as a percentage of baseline income, logged deflated transfer amounts, programme provision of medical care, provision of nutritional supplements, provision of behaviour change communication (overall and by type of BCC, including nutritional, medical, washing/sanitation/hygiene (WASH), and infant and child education, as well as population characteristics taken from the World Development Indicators including anaemia rates among mothers and children (separately). Full results are in the supplement; only a few are significantly associated with either haemoglobin levels or anaemia prevalence, as shown in Table 2. World Development Indicators (WDI) prevalence levels of anaemia among children and transfer size as measured several different ways (i.e. deflated log of total transfers and amounts as a percentage of baseline income) are never significant.

**Table 2:** Meta‐regression results.

Programme provides	Impact on haemoglobin (g/dL)	Impact on anaemia prevalence
Nutritional supplements	0.41*** CI (0.21, 0.61) (*N* = 10)	−6.9** CI (−13.5, −0.36) (*N* = 13)
Access to health care	0.25** CI (0.05, 0.45) (*N* = 12)	0.38 CI (−3.09, 3.86) (*N* = 14)
WDI anaemia prevalence among mothers	−0.002 CI (−0.01, 0.01) (12)	−0.43* CI (−0.90, 0.04) (15)

*N* in parentheses.

* Significant at the 10% level.

** Significant at the 5% level.

*** Significant at the 1% level. Full results in Appendix Table A3.

The only programme characteristic associated with anaemia prevalence is the provision of supplements. Note that haemoglobin improvements are weakly linked to both nutritional supplements and to access to health care. The average impact on haemoglobin and anaemia is apparently insensitive to the total amount of the transfer: Appendix Table A3 shows that the meta‐regression results with haemoglobin as the dependent variable give the expected sign (i.e. more cash is associated with more haemoglobin), but the coefficients are insignificant, with *p* > 0.75 in both cases. A meta‐regression of anaemia prevalence on the percent of household income received in a cash transfer programme yields a small positive coefficient, implying that more cash is not effective; the outcome is insignificant with a *p*‐value over 0.5. There is not enough data to investigate associations of cash equivalent transfer amounts with anaemia.

**Figure 3:**
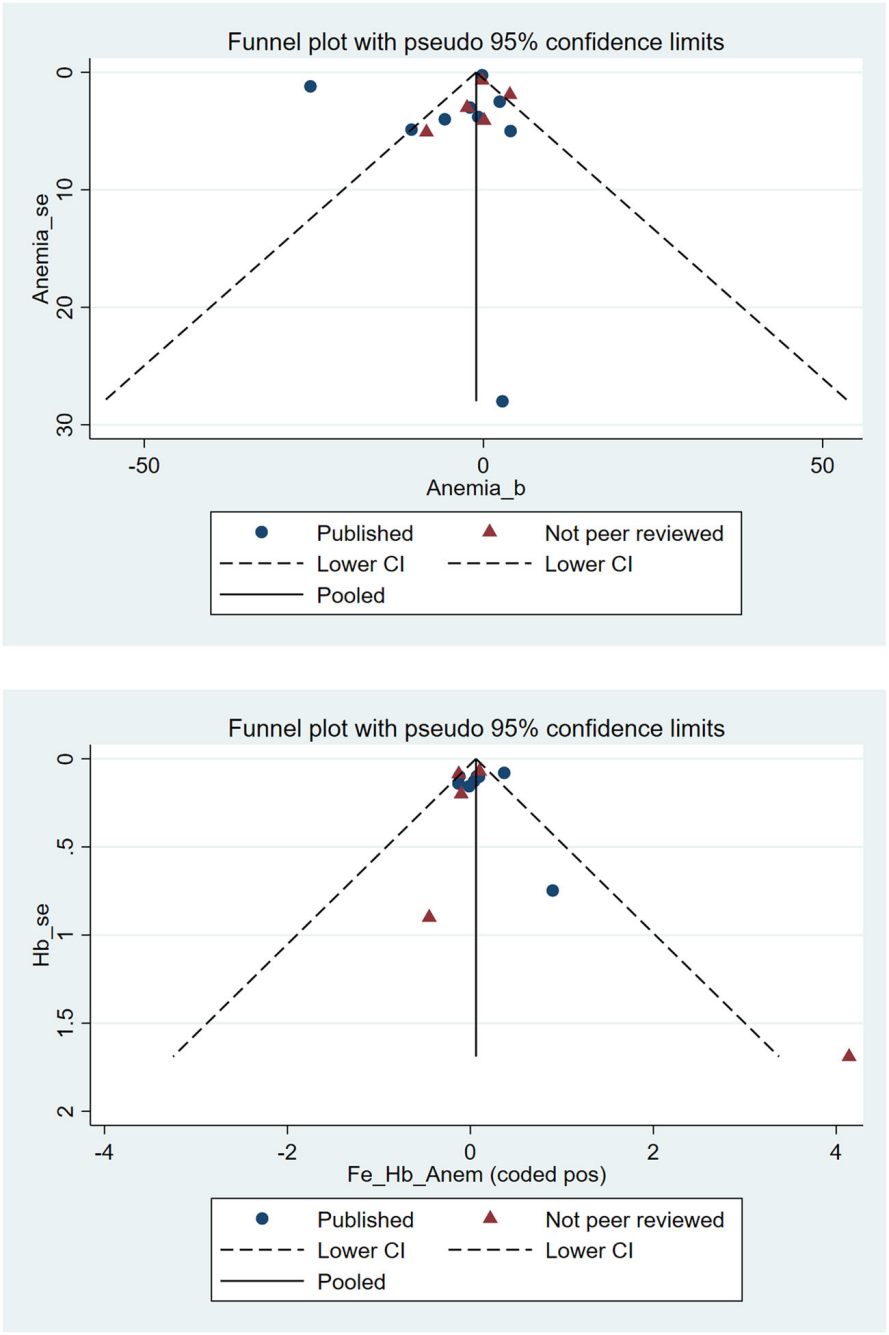
Meta‐funnel plots checking for publication bias.

Figure 3 shows two meta‐funnel plots, which are designed to contrast studies finding significant results from those showing insignificant results. Studies below the dashed lines show a ratio of estimated impact to standard error lower than the level commonly associated with statistical significance. Here we see no evidence of publication bias, as the icons representing peer‐reviewed studies and non‐peer‐reviewed studies are dispersed together.

## 4 Discussion

The current paper finds that cash assistance alone is not strongly associated with improved haemoglobin status. In partial contrast, improvements in haemoglobin were much more common in programmes that provided micronutrient supplements in tandem. Considering only programmes that do not provide supplements, our combined estimates are tantamount to a null result.

Manley et al. (2020, 2022) found that cash transfer programmes improve dietary diversity and the consumption of animal‐source foods. In principle, such dietary changes could provide a plausible pathway to improved haemoglobin levels and decreased anaemia. The income transfers could also lead to greater access to health care, thereby influencing child anaemia through pathways that are not directly linked to iron intake. Moreover, since nutrition‐sensitive social protection programmes affect all household members, the pathways of dietary diversity and increased consumption of animal‐source foods can influence children both directly through their own diets and possibly through the quality of their mother's diet as well as through access to antenatal care and other health services. Indeed, one study has shown that a cash transfer in Brazil was associated with reduced malaria although it did not report on anaemia (Alves et al. 2021). Thus, an indirect contribution of transfers to child anaemia and Hb via maternal health is an additional plausible expectation.

The fact that the programmes studied did not have a measurable impact might reflect the size of the transfers, especially when one considers the gap between the incomes of many recipients and the cost of a healthy diet (FAO IFAD UNICEF WFP WHO 2022). The null results are also consistent with Alderman and Linnemayr (2009), which reported that anaemia responded only modestly to higher income. Their study was based on an approach which considered all forms of anaemia, not just iron deficiency anaemia, as in most of the studies included in the current analysis. Among households who already have some level of iron in their diet, the increase mediated by the income transfer may not be enough to matter. Similarly, if the role of iron is not the primary causal factor in child anaemia, the additional purchasing power by itself may not reduce morbidity sufficiently to lead to measurable impacts on anaemia prevalence.

Ruel and Alderman (2013) and subsequent studies of nutrition‐sensitive social protection draw a distinction between such programmes and conventional transfer programmes. In addition to income support which is the main objective of transfer programmes, the former include design features such as: (1) targeting the most nutritionally vulnerable members within poor households such as pregnant and lactating women or young children; (2) linkages to nutrition specific interventions including behavioural change communication or the provision of fortified foods; and (3) inclusion of specific nutrition outcomes which are monitored. Moreover, some transfers include conditionalities to encourage utilization of health services.

One limitation of this study is that we are unable to disentangle the contribution of cash per se from such design features particularly the provision of iron and micronutrient supplements. Since many cash transfer programmes also provide supplements to either mothers or to children, it is not immediately clear whether the improvements in haemoglobin or anaemia status are attributable more to the supplements provided or to the improved diet. Mexico's transfer programme is a case in point; Ramírez‐Silva et al. (2013) find indications that the impacts of the Mexican cash transfer programme were attributable predominantly to the initiative's provision of nutritional supplementation with the cash, but it is not clear whether that outcome is generalizable.

Andrew et al. (2016) find that even micronutrient supplements were not effective in their sample, perhaps due to the fact that anaemia among older children may be less related to iron deficiency. If the change in diet is inframarginal with respect to iron, or, than the impacts on haemoglobin/anaemia might not result. Andrew et al.'s (2016) meta‐analysis finds considerable heterogeneity in effectiveness: different conditions require different solutions, even among supplements. The story for cash transfers may be unfortunately similar to the analysis of iron fortification (Qureshy et al. 2023) which argued that the confidence intervals in the diverse studies reviewed were so wide that any conclusion on programme efficacy was difficult.

Another key limitation of the study is that it was only able to locate a modest set of papers indicating the impact of transfer programmes on child anaemia. This limitation also hindered the investigation of the interplay of transfers and supplements. Ideally, we would have wanted to include 2 by 2 analyses with arms covering interventions with supplements, with cash, and with both jointly. There are a few studies with such a design looking at stunting, for example Soofi et al. (2022) but to our knowledge, none covering anaemia. Similarly, the limited evidence base was not sufficient to ascertain other design features, including the size of transfers, on outcomes.

This point is the basis for our main conclusion. Given the popularity of transfer programmes and the extent of anaemia, it is surprising that relatively few studies of nutrition sensitive social protection have included outcome of transfers on either Hb or anaemia. The meta‐review of Moorthy et al. (2020) concludes that supplements are a preferred intervention against anaemia, and our evidence hints that cash in concert with supplements may be more effective than cash alone. Complementarities of cash and supplements may exist, but at this time the possibility remains speculative.

## Author Contributions

J.M. designed the study and performed the analysis with guidance from H.A. Both contributed to writing the paper, and both have read and approved the final manuscript.

## Conflicts of Interest

The authors declare no conflicts of interest.

## Figures and Tables

**Figure A1:**
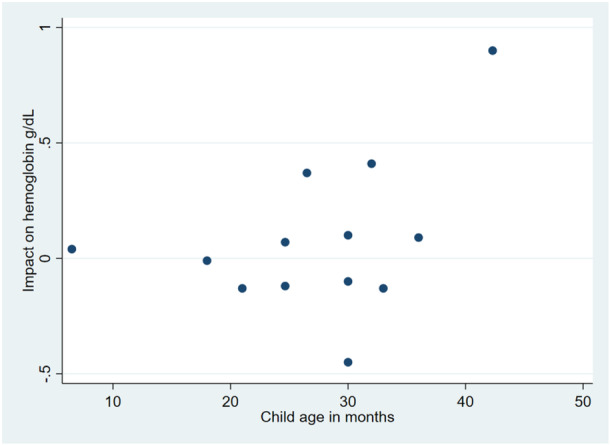
The overall impacts graphed with the age of the sample. Most surveys tracking haemoglobin impacts cover children with an average age under 36 months. Haemoglobin versus average age of children in sample.

**Figure A2:**
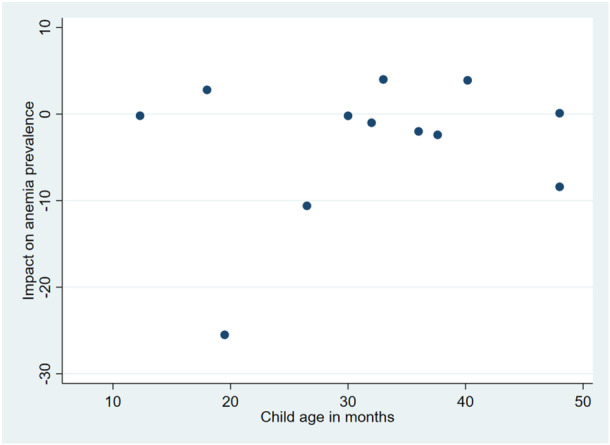
Programme impact on anaemia status by age.

**Figure A3:**
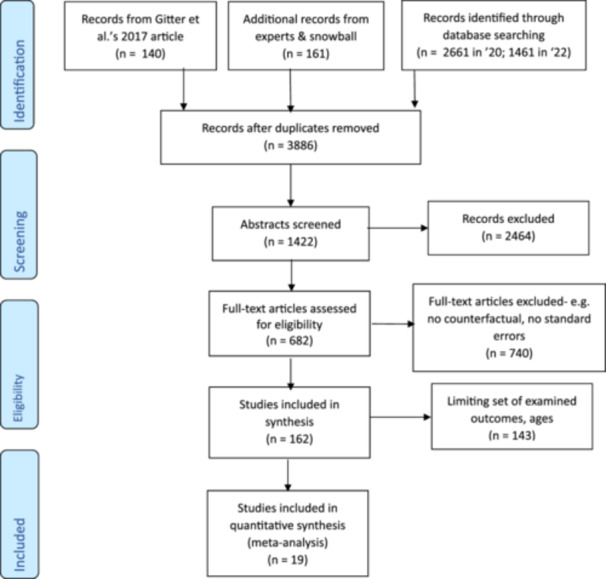
PRISMA diagram describing the search process.

## Data Availability

The only data used was publicly available, that is all numbers are from other papers.

## Endnotes

1 We found one study focusing on 10‐year‐olds (Behrman et al. 2008), two estimates on adolescent females from Baird et al. (2019), and results on mothers from Schady (2012), Newton‐Lewis and Viswanathan (2017), and Mathur and Sen (2024). For consistency we used two of the estimates from Gilligan et al. (2013) for younger children and left out their estimate for the oldest age group. Also for consistency we chose not to include Gertler (2004) as the differences in prevalence rates in the table {48.3 and 41.0] differs from the claim reported on page 340 of his article. ‘The results show that treatment children are 25.5 percent less likely to be anaemic, and this difference is statistically significant at the 1‐percent level.’ We have chosen to note this in the appendix but to exclude this in the main body.

## 1

See Tables A1, A2, A3.

**Table A1:** Impact estimates with confidence intervals.

Listing	Country	RCT?	Sample size	Writeup year	Child ages	Hb impact (g/dL)	CI (Hb)	Baseline Hb	Anaemia prevalence	CI (anaemia)	Baseline anaemia
Avitabile et al. (2019)	Mexico	RCT	2403	2019	0–72 months (baseline)				−2.40	(−8.28, 3.48)	0.190
Benedetti et al. (2016)	Honduras	RCT	1791	2016	0–3 years at baseline				2.80	(−2.69, 8.29)	0.449
Celhay et al. (2016)	Bolivia	obs	3820	2018	24–60 months				−5.70	(−13.54, 2.14)	0.616
Cunha (2014)	Mexico	RCT	2449	2014	2–6 years at followup				−2.00	(−7.88, 3.88)	0.210
Fenn et al. (2017), double cash	Pakistan	RCT	6141	2017	6–48 months (baseline)	0.07	(−0.12, 0.26)	9.00			
Fenn et al. (2017), single cash	Pakistan	RCT	6141	2017	6–48 months (baseline)	−0.12	(−0.31, 0.07)	8.90			
Fernald and Hidrobo (2011)	Ecuador	RCT	1196	2011	12–35 months	0.04	(−0.21, 0.29)	9.60			
Fernald et al. (2008)	Mexico	RCT	2449	2008	24–72 months	0.90	(−0.57, 2.36)	11.85			
Gajate‐Garrido (2014)	Peru	obs	4602	2014	6–59 months	0.41	(0.08, 0.75)	11.10	−1.00	(−13.72, 13.72)	0.346
Gilligan et al. (2013)	Uganda	RCT	612	2013	36–53 months				−8.40	(−18.4, 1.6)	N/A
Gilligan et al. (2013)	Uganda	RCT	898	2013	6–35 months				0.10	(−7.94, 8.14)	N/A
Hidrobo et al. (2012)	Colombia	RCT	785	2012	6–59 months	−0.13	(−0.4, 0.14)	10.85	4.00	(−5.8, 13.8)	0.480
IFPRI International Food Policy Research Institute (2003)	Honduras	RCT	2908	2003	12–23 months	0.10	(−0.04, 0.24)	11.20			
Maluccio and Flores (2004)	Nicaragua	RCT	963	2004	6–59 months	−0.10	(−0.49, 0.29)	11.45	−0.20	(−1.53, 1.13)	0.326
McIntosh and Zeitlin (2018) (L)	Rwanda	RCT	2372	2018	< 60 months				−0.78	(−8.23, 6.67)	0.180
McIntosh and Zeitlin (2018) (S)	Rwanda	RCT	2372	2018	< 60 months				2.40	(−2.5, 7.3)	0.180
Paxson and Schady (2010)	Ecuador	RCT	1654	2010	3–7 years at followup	0.09	(−0.11, 0.29)	10.34			
Perova and Vakis (2009)	Peru	obs	128	2009	< 60 months	−0.45	(−2.21, 1.31)	11.45			
Rivera et al. (2004)	Mexico	RCT	650	2004	< 12 months at baseline	0.37	(0.21, 0.53)	10.75	10.60	(1.04, 20.16)	0.443
von Fenn et al. (2017)	India	obs	12523	2021	1–5 years at interview				3.90	(0.18, 7.62)	0.475
Younger et al. (2009)	Ecuador	RCT	2615	2009	6–36 months (baseline)	−0.13	(−0.3, 0.04)	10.08			
Zhou et al. (2020)	China	obs	1522	2020	Avg age 12 months				−0.19	(−0.68, 0.3)	N/A

**Table A2:** Programme details.

Listing	Programme	Country	Total years	Transfers/year	USD/year	Educ condition?	Health care condition?	Health care provided?	Supplements?	BCC/visits?
Avitabile 19	PAL: Programa de Apoyo Alimentario	Mexico	2	6	225	no	no	no	no	no
Benedetti et al. (2016)	Bono 10,000	Honduras	1	3	342	yes	yes	yes	no	no
Celhay et al. (2016)	Bono Juana Azurduy	Bolivia	3	6	95	no	yes	yes	yes	yes
Cunha (2014)	PAL: Programa de Apoyo Alimentario	Mexico	2	6	180	no	no	no	no	yes
Fenn et al. (2017), double cash	Action Against Hunger	Pakistan	1	12	168	no	no	no	no	no
Fenn et al. (2017), single cash	Action Against Hunger	Pakistan	1	12	168	no	no	no	no	no
Fernald and Hidrobo (2011)	Bono de Desarrollo Humano	Ecuador	2	12	180	no	no	no	no	no
Fernald et al. (2008)	PROGRESA/Oportunidades/Prospera	Mexico	5	6	200	yes	yes	yes	yes	yes
Gajate‐Garrido (2014)	Juntos	Peru	4	12	444	yes	yes	yes	no	no
Gilligan et al. (2013): 36–53 months	Karamoja cash transfer pilot	Uganda	1	9	89	no	no	no	no	no
Gilligan et al. (2013): 6–35 months	Karamoja cash transfer pilot	Uganda	1	9	89	no	no	no	no	no
Hidrobo et al. (2012)	Food, Cash, & Voucher (WFP)	Colombia	1	12	240	no	no	no	no	yes
IFPRI International Food Policy Research Institute (2003)	Programa de Asignacion Familiar	Honduras	2	12	18	yes	yes	yes	no	no
Maluccio and Flores (2004)	Red de Proteccion Social	Nicaragua	2	6	296	yes	yes	yes	no	yes
McIntosh and Zeitlin (2018) (L)	Gikuriro	Rwanda	1	12	555	no	no	no	no	no
McIntosh and Zeitlin (2018) (S)	Gikuriro	Rwanda	1	12	555	no	no	no	no	no
Paxson and Schady (2010)	Bono de Desarrollo Humano	Ecuador	2	12	180	no	no	yes	no	no
Perova and Vakis (2009)	Juntos	Peru	2	12	360	yes	yes	yes	yes	no
Rivera et al. (2004)	PROGRESA/Oportunidades/Prospera	Mexico	2	6	300	yes	yes	yes	yes	yes
von Haaren and Klonner (2020)	Indira Ghandhi Matritva Sahyog Yojana	India	1	3	74	no	yes	yes	no	yes
Younger et al. (2009)	Bono de Desarrollo Humano	Ecuador	2	12	144	no	no	no	no	no
Zhou et al. (2020)	Maternal & Child Health Conditional Cash Transfer	China	1	6	154	no	yes	yes	no	no

**Table A3:** Results of all meta‐regressions.

Dependent: Explanatory	Haemoglobin (g/dL)	*N*	Anaemia prevalence	*N*
WDI maternal anaemia	−0.003 (−0.014, 0.008)	12	−0.430 (−0.901, 0.041)	9
WDI child anaemia	−0.003 (−0.016, 0.010)	12	−0.135 (−0.391, 0.121)	9
Transfer as share of baseline household income	0.002 (−0.014, 0.018)	9	0.037 (−0.114, 0.187)	8
Log of deflated yearly transfer	0.040 (−0.126, 0.205)	11	N/A (too few observations)	
Programme provides medical care	0.247 (0.045, 0.450)	12	0.383 (−3.092, 3.859)	14
Programme provides nutritional supplements	0.410 (0.207, 0.613)	10	−6.941 (−13.523, −0.360)	13
Programme provides behaviour change communication (BCC)	0.078 (−0.229, 0.386)	12	−0.316 (−3.803, 3.171)	14
BCC focus includes infant and young children feeding	0.164 (−0.260, 0.588)	8	0.849 (−3.587, 5.284)	10
BCC focus includes general nutrition information	0.164 (−0.260, 0.588)	8	0.458 (−4.319, 5.235)	10
BCC focus includes healthcare	0.164 (−0.260, 0.588)	8	0.527 (−4.580, 5.633)	10
BCC focus includes WASH (sanitation & hygiene)	0.164 (−0.260, 0.588)	8	−1.971 (−6.961, 3.019)	10

Confidence intervals reported below‐estimated impacts.

The last four estimates under haemoglobin (the four types of BCC) are identical, as the programmes in the sample provided all the same types of BCC.

One result we decided to exclude based on the chosen methods of analysis is Gertler (2004). Gertler interprets his logistic regression results as showing that cash is associated with a 25.5% drop in anaemia prevalence, a change in probability 3.5× more than the listed difference in prevalence between the treatment and control groups according to his Table 3. This change is due to the nature of the log function that is being used, and is qualitatively different from the means of analysis deployed in the other studies in our sample. If we use the unconditional difference in means of 7.3% (still third highest of our 14 estimates) and we keep the *p*‐value listed in his Table 3, the meta‐analysis shows an average change in anaemia prevalence from cash of 0.26 percentage points with a *p*‐value of 0.68. In short, it does not change our conclusions.

See Figures A1, A2, A3.
